# 425. Characterization and predictive risk scoring of long COVID in a South Indian cohort after breakthrough COVID infection; a prospective single centre study

**DOI:** 10.1093/ofid/ofad500.495

**Published:** 2023-11-27

**Authors:** Merlin Moni, Pranav Nair, Chithira Nair, Kiran Kulirankal, Elizabeth Corley, Fabia Edathadathil, Georg Gutajhr, Dipu T Sathyapalan

**Affiliations:** Amrita Institute of Medical Sciences, Kochi, Kochi, Kerala, India; Amrita hospital, kochi, Ernakulam, Kerala, India; Amrita Hospital, Kochi, Ernakulam, Kerala, India; Amrita Hospital,Kochi, Ernakulam, Kerala, India; Weill Cornell Medicine, New York, New York; Amrita Institute of Medical Sciences and Research Center, kochi, Kerala, India; Amrita Vishwa Vidyapeetham, Kollam, Kerala, India; Amrita Hospital,Kochi, Ernakulam, Kerala, India

## Abstract

**Background:**

The majority of existing literature on long COVID has been focused on hospitalised patients. However, in the real-world setting of the post-vaccination era where breakthrough infections are the norm, it becomes a priority to assess the clinical profile of long COVID symptoms among such individuals. Through our study, we aim to describe the incidence, characterise and stratify the risk of developing long COVID breakthrough infections.

**Methods:**

This prospective observational study included adult patients with breakthrough COVID-19 infections diagnosed at a tertiary hospital in India. Post-COVID symptoms at weeks 2, 6 and 12 after testing negative were extracted using a questionnaire after which multi-variate analysis was done.

**Results:**

Out of 414 patients analysed, 164 reported post-COVID symptoms beyond 6 weeks of the infection. Univariate analysis showed that the presence of long COVID was found to be significantly higher among patients above 65 years of age at 31.3% in comparison to the long COVID presence at 29.4% among those below age 65 (p < 0.001). Systemic hypertension was also significantly associated with the presence of long COVID (72.3%), along with bronchial asthma (68.8%) with a p-value of < 0.0001 in both instances. Backwards selection was used leading to a reduced model consisting of age OR 1.053, 95% CI 0.097–1.07), p < 0.001), hypertension (OR 2.59, 95% CI 1.46–4.59, p = 0.001) and bronchial asthma (OR 3.7176, 95% CI 1.24–11.12, p = 0.018) to be significant predictors of long COVID incidence. These were used to develop a propensity score (C statistics – 80.9%).

**Figure:**
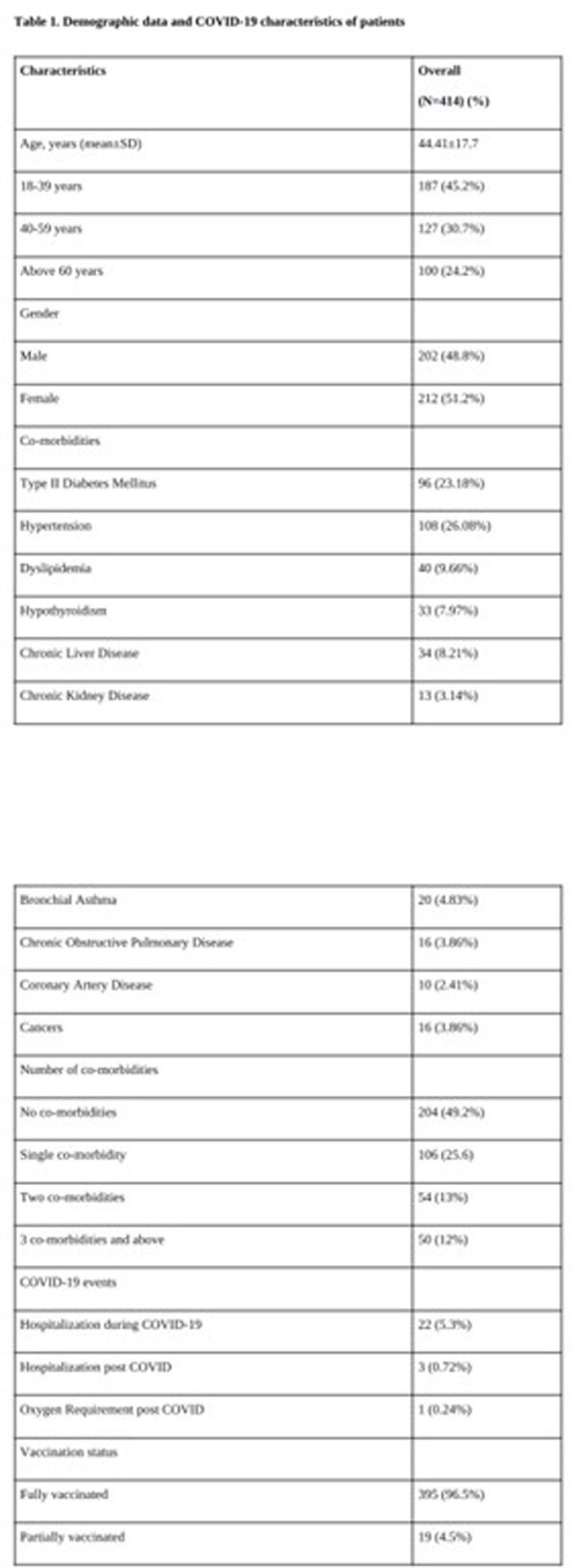

**Figure:**
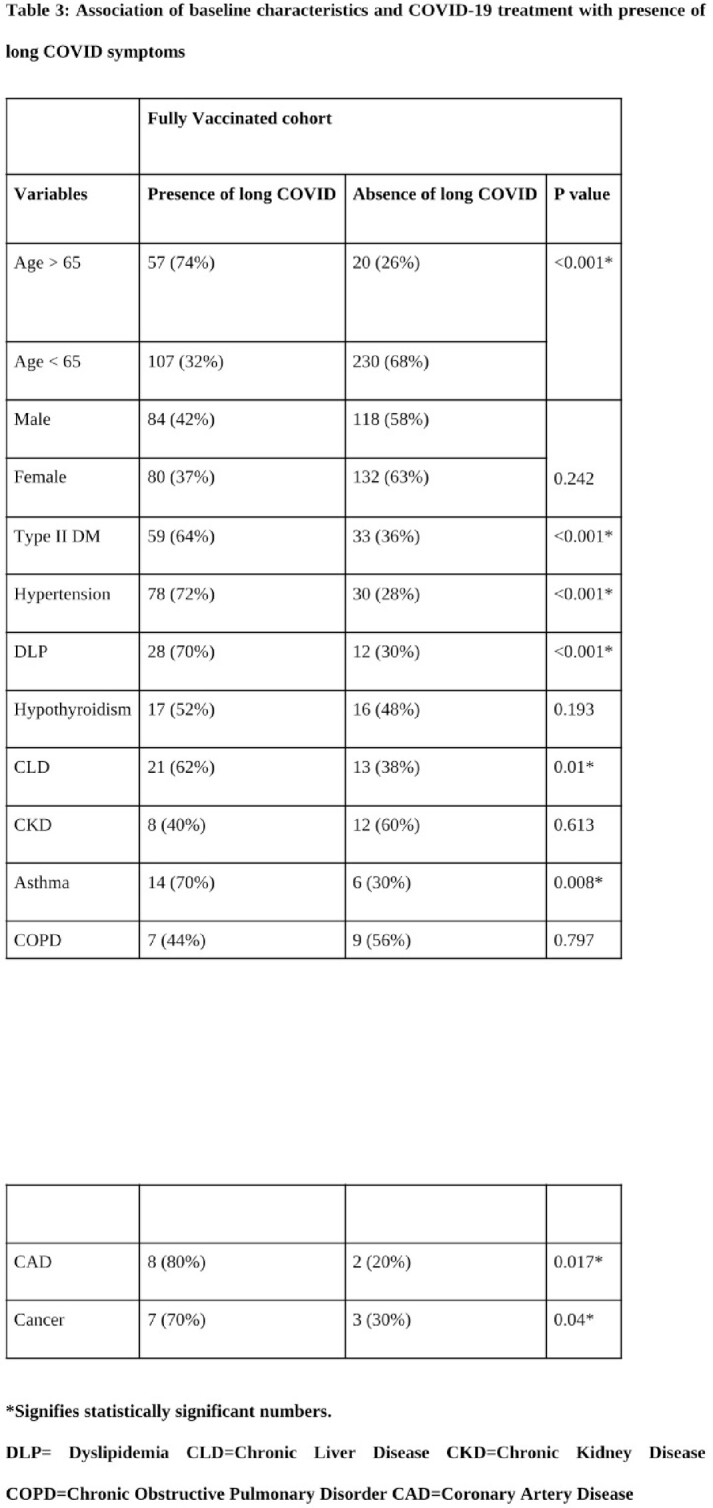

**Figure:**
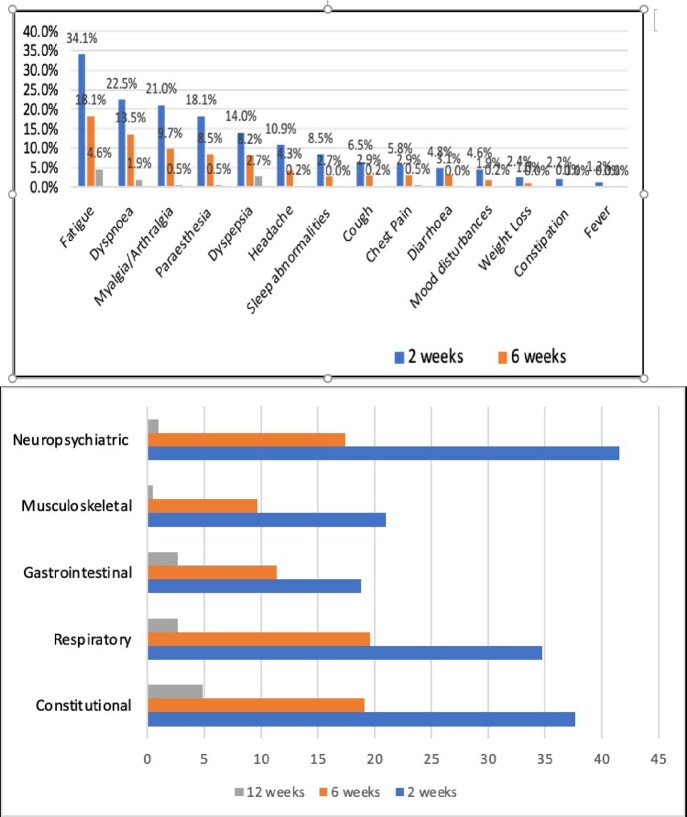

1A: Long COVID symptomatic profile at weeks 2, 6 and 12 weeks 1B: Organ system wise distribution of long COVID symptoms at 2, 6 and 12 weeks

**Conclusion:**

A significant presence of long COVID at 12 weeks among non-hospitalised breakthrough infections calls for a series of review check-ups for the early detection of long-term complications. The proposed predictive risk scoring based on significant risk factors may assist clinicians in identifying patients at risk of developing long COVID, leading to appropriate, individualised management.

Table 4

**Figure:**
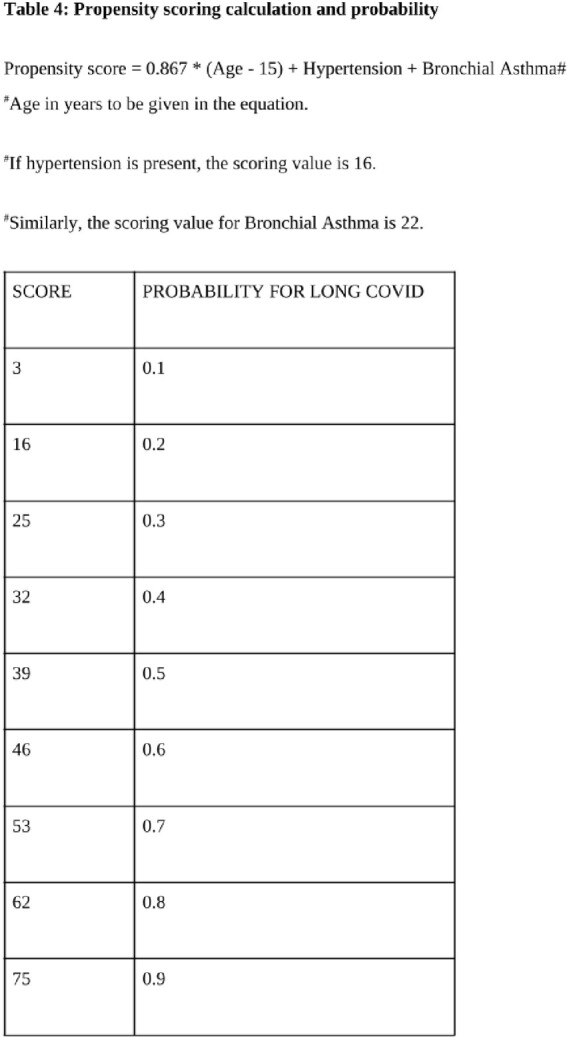

Figure 3A & 3B

**Figure:**
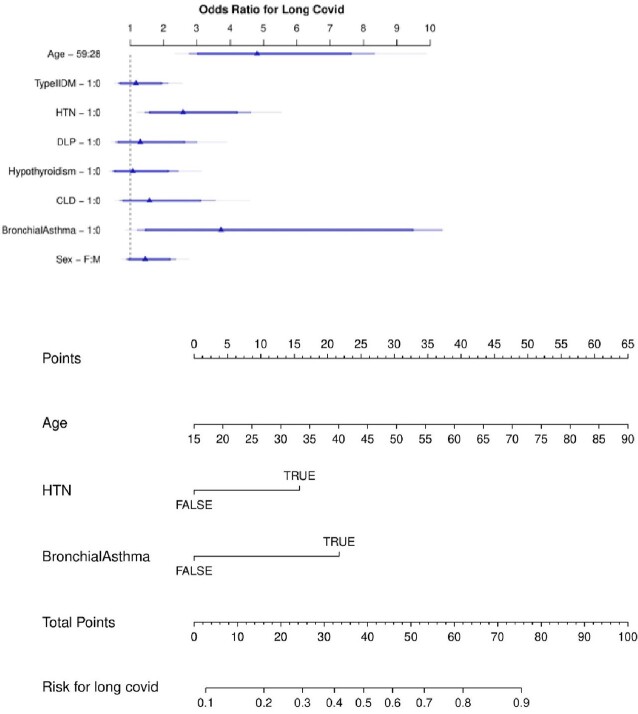

3A: Logistic regression model for predicting long COVID incidence 3B: Clinical prediction nomogram to depict risk of long COVID incidence

**Figure 4A & 4B**

**Figure:**
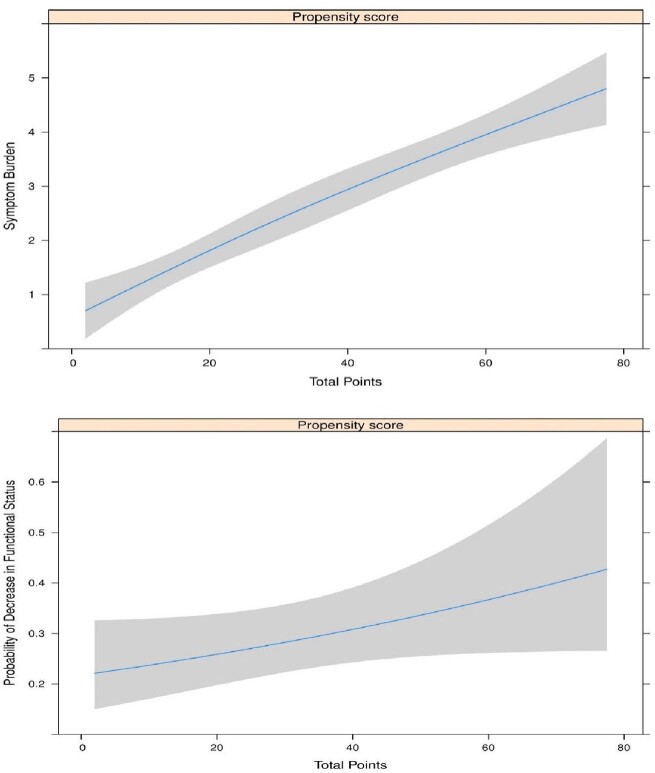

4A: Association of propensity risk score for Long COVID incidence with symptom burden 4B: Association of propensity risk score for Long COVID incidence with functional status

**Disclosures:**

**All Authors**: No reported disclosures

